# The different clonal origins of metachronous and synchronous metastases

**DOI:** 10.1007/s00432-023-05007-3

**Published:** 2023-06-20

**Authors:** Ofer N. Gofrit, Ben Gofrit, Yuval Roditi, Aron Popovtzer, Steve Frank, Jacob Sosna, Marina Orevi, S. Nahum Goldberg

**Affiliations:** 1 Department of Urology, Faculty of Medicine, Hadassah Medical Center, Hebrew University of Jerusalem, P.O.B 12000, 91120 Jerusalem, Israel; 2 School of Engineering and Computer Science, Hebrew University of Jerusalem, Jerusalem, Israel; 3 Department of Oncology, Faculty of Medicine, Hadassah Medical Center, Hebrew University of Jerusalem, Jerusalem, Israel; 4 Department of Radiology, Faculty of Medicine, Hadassah Medical Center, Hebrew University of Jerusalem, Jerusalem, Israel; 5 Department of Nuclear Medicine, Faculty of Medicine, Hadassah Medical Center, Hebrew University of Jerusalem, Jerusalem, Israel

**Keywords:** Metachronous metastases, Synchronous metastases, Clonal origin, Linear, Parallel ratio

## Abstract

**Background:**

Metastases are the leading cause of mortality in cancer patients. Linear and parallel are the two prominent models of metastatic progression. Metastases can be detected synchronously along with the primary tumor or metachronously, following treatment of localized disease. The aim of the study was to determine whether synchronous metastases (SM) and metachronous metastases (MM) differ only in lead-time or stem from different biological processes.

**Materials and methods:**

We retrospectively studied the chest CTs of 791 patients inflicted by eleven malignancy types that were treated in our institution in the years 2010–2020. Patient’s population included 396 with SM and 395 with MM. The diameter of 15,427 lung metastases was measured. Clonal origin was deduced from the linear/parallel ratio (LPR)-a computerized analysis of metastases diameters. LPR of  1 suggests pure linear dissemination and − 1 pure parallel.

**Results:**

Patients with MM were significantly older (average of 62.9 vs 60.7 years, *p* = 0.02), and higher percentage of them were males (58.7% vs 51.1%, *p* = 0.03). Median overall survival of patients with MM and SM was remarkably similar (23 months and 26 months respectively, *p* = 0.774) when calculated from the time of metastases diagnosis. Parallel dissemination (LPR ≤ 0) was found in 35.4% of patients with MM compared to only 19.8% of the patients with SM (*p* < 0.00001).

**Conclusion:**

Patients with SM and MM differ in demography and in clonal origin. Different therapeutic approaches may be considered in these two conditions.

**Supplementary Information:**

The online version contains supplementary material available at 10.1007/s00432-023-05007-3.

## Introduction

Cancer is an extremely complex and diverse disease. Yet there are a few traits common to almost all tumors, justifying their investigation as a combined entity. Invasion and metastasis formation are the defining features of malignancy, with metastases responsible for 90% of cancer related deaths (Fouad and Aanei 2017). Metastases can be discovered either upon the initial diagnosis of cancer-synchronous metastases (SM) or during follow-up after treatment of the localized disease-metachronous metastases (MM). Comparisons of the clinical course, radiological findings, and genomics of SM and MM can potentially provide clinically meaningful information on both processes.

It is expected that MM that are actively detected during follow-up programs will be smaller, fewer and with better prognosis compared to SM. Unfortunately, the literature comparing SM and MM is retrospective and the reports are conflicting and often contradictory. In colorectal cancer some manuscripts report on similar prognosis of patients with SM or MM (Slesser et al. 2013; Mitry et al. 2010; Bokhorn et al. 2008; Nozawa et al. 2012; Jeong et al. 2017; Tsai et al. 2007; Mekenkamp et al. 2010), while others report on slightly better prognosis of patients with MM (Reboux et al. 2022; Kumar et al. 2014; Colloca et al. 2020; Ghiringhelli et al. 2014). In breast cancer, both better and similar prognosis of patients with MM have been reported (Dawood et al. 2010; Khanfir et al. 2013). This disagreement is partially due to the absence of an accepted single definition of SM and MM and different authors place the cutoff at various time points, from 0 to 12 months after the initial diagnosis.

**Fig. 1:**
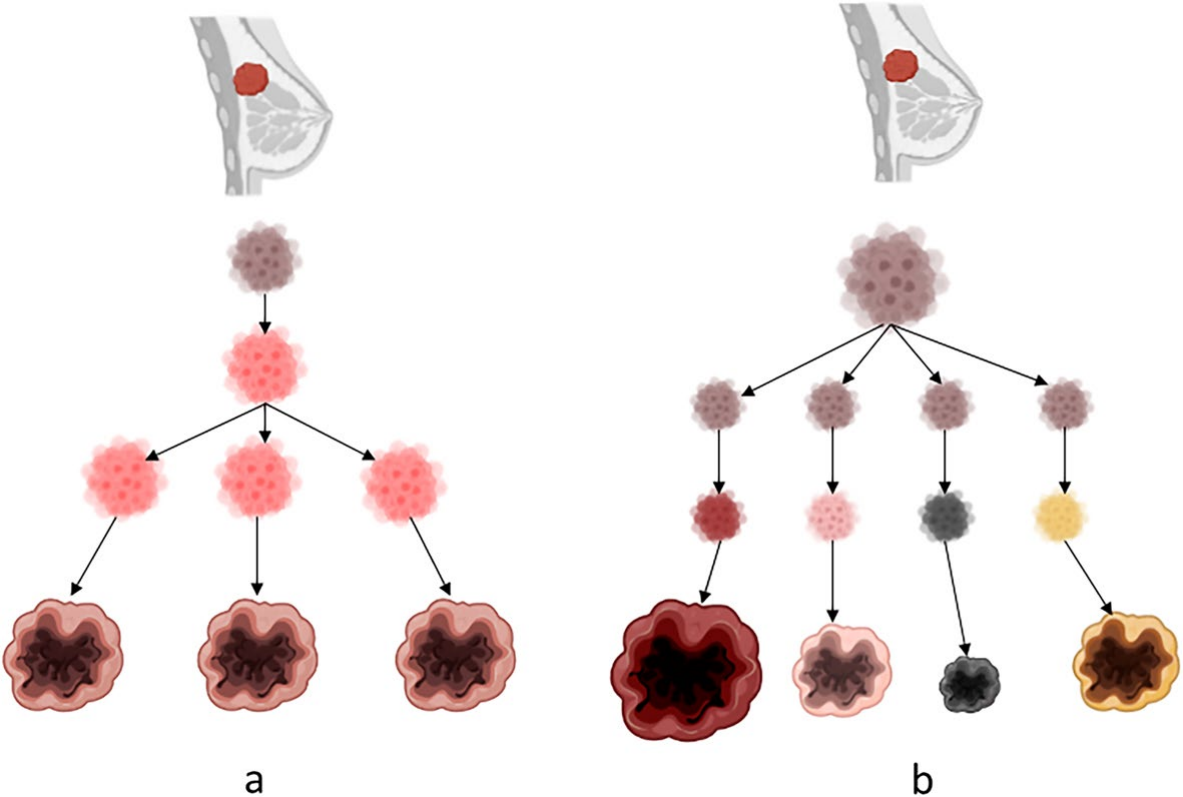
**a** The linear model of metastases dissemination. After accumulating the necessary capabilities for metastatic spread inside the primary tumor, a wave of metastases is deployed. These cells bare high resemblance to the primary tumor and to each other. Further waves can be deployed when additional clones reach this maturity. **b** The parallel model of metastatic spread. There is preclinical spread of disseminated tumor cells (DTCs) that continue to evolve independently in target organs and generate metastases. High disparity between them and the primary tumor and among them is expected

Linear and parallel are the two leading models of metastatic progression Fig. 1 (Stoecklein and Klein 2010). The linear model assumes accumulation of genetic and epigenetic alterations within the primary tumor and dissemination of fully malignant waves of cells. These cells bear a high resemblance to the primary tumor and to each other. The parallel model suggests early, preclinical spread of less advanced disseminated tumor cells (DTCs). These cells evolve independently in the target organs and generate highly diverse metastases. These variations in clonality will have major implications for medical oncologic therapy.

In this study, we compared the demographics, radiological findings, and outcome of patients with SM and MM. We traced metastases clonal evolution using a simple method that can quantitatively differentiate between linear and parallel spread (Gofrit et al. 2022). The method is based on the logical assumption that cells originating from the same clone, that left the primary tumor at the same time, and implanted in the same organ (such as the lungs studied here) will grow at a similar pace and reach a similar diameter at any time point. These metastases can be clustered into several groups with similar diameter. On the other hand, parallel spreading metastases that are genetically diverse are expected to show high variability of diameters and cannot be clustered according to diameter.

## Materials and methods

### Patients

The study included 791 patients with 15,427 lung metastases originating in malignancies of the bladder, breast, colorectum (CR), Ewing sarcoma, kidney, melanoma, pancreas, prostate, adult sarcomas, stomach, and thyroid, that were treated in our institutions in the years 2010–2020. The records of these patients and their chest CTSs were retrospectively reviewed, and the following parameters recorded: age, gender, date of cancer diagnosis, date of metastasis diagnosis, overall survival, metastases number, and largest diameter of each metastasis on patient’s latest axial chest CT. Earlier CT studies were analyzed when patient’s latest CT was difficult to interpret due to large pleural effusion or coalescent of several metastases. This study was conducted in accordance with the Declaration of Helsinki. Being a retrospective study based on medical chart review and standard follow-up data informed consent was waived by the Hadassah Medical Organization (HMO) IRB (# 0650-21). All experiment protocols were approved by the Hadassah Medical Organization (HMO) IRB (# 0650-21). Patients with history of primary lung cancer or patients diagnosed with more than one malignancy type were excluded from the study.

### Statistical analysis

SM was defined as metastases detected prior to or at the time of cancer diagnosis and MM as metastases diagnosed at any time later. The raw data generated and analyzed during the current study are available in supplementary material S1.

The linear/parallel ratio (LPR) measures the ratio between lung metastases that can be clustered and metastases that cannot be clustered. LPR was calculated for each patient by arranging all metastases in a rising order of diameters and grouping all metastases with similar diameter (with diameter difference ≤ 1 mm on axial chest CT) into a single cluster. The LPR was then calculated with the aid of a computer code (available in supplementary material S2) using the formula: LPR = (∑*c* − ∑*i*)/(∑*c* + ∑*i*) where “c” is the sum number of metastases that can be clustered metastases and “i” the sum number of isolated metastases (Liang et al. 2021). LPR ≤ 0 suggests dominance of the parallel spreading pathway and LPR > 0 suggests dominance of the linear pathway.

Overall survival (OS) was deduced from Kaplan–Meier plots using the log-rank method. Student *t*-test and Fisher exact tests were employed when appropriate.

## Results

791 patients with 15,427 lung metastases, including 395 patients with lung MM and 396 with lung SM were studied. A breakdown according tumor types is presented in Table 1. The median lead-time from diagnosis of the primary tumor to MM was 27 months (IQR 12, 27). Lead-time was shortest for carcinoma of the stomach (median of 12.5 months, IQR 6.75, 22) and the longest for prostate cancer (median of 48 months, IQR 35.5, 60 months). Metastases to sites other than the lungs were found in 224 patients with MM (56.7%) and 283 patients with SM (71.4%).

**Table 1:** Number of patients with synchronous and metachronous lung metastases and median lead-time from initial diagnosis to diagnosis metastases in patients with metachronous disease

Type	Synchronous	Metachronous	Median lead-time in months (IQR)	Total
Bladder	18 (34.6%)	34 (65.4%)	22 (6.25, 42.25)	52
Breast	53 (49.0%)	55 (51.0%)	56 (24,120)	108
Colorectal	128 (53.3%)	112 (46.7%)	24 (15, 40.5)	240
Ewing	10 (45.4%)	12 (54.6%)	14 (8.75, 38)	22
Kidney	27 (39.1%)	42 (60.9%)	36 (12, 84)	69
Melanoma	28 (35.9%)	50 (64.1%)	24 (11.5, 21.5)	78
Pancreas	40 (93.0%)	3 (7.0%)	22.5 (12.75, 17)	43
Prostate	21 (64.7%)	13 (35.3%)	48 (35.5, 60)	34
Sarcoma	34 (42.5%)	46 (57.5%)	14 (6, 13)	80
Stomach	12 (72.2%)	6 (27.8%)	12.5 (6.75, 22)	18
Thyroid	25 (53.2%)	22 (46.8%)	40 (14, 34)	47
Total	396 (50.6%)	395 (49.3%)	27 (12, 27)	791

Demographic and radiographic parameters of the entire cohort are presented in Table 2. These parameters classified by cancer type are shown in supplementary material S3. Patients with MM and SM were similar in age at the time of cancer diagnosis, but MM patients were on average 26 months older when metastases were diagnosed (*p* = 0.02). This difference was most prominent in carcinomas of the breast (average age difference of 75 months, *p* = 0.04), CR (average age difference of 78 months, *p* = 0.0006), and thyroid (average age difference of 126 months, *p* = 0.02). There was a significantly higher percentage of men among patients with MM compared to patients with SM (58.7% vs 51.1%, *p* = 0.03).

**Table 2:** Demographic and radiographic parameters of patients with synchronous and metachronous lung metastases

Parameter	Synchronous	Metachronous	Total	*p*
Average age at diagnosis of cancer(SD)	60.7 (16.7)	59.3 (16.9)	59.7 (16.8)	0.514*
Average age at diagnosis of metastases (SD)	60.7 (16.7)	62.9 (17.0)	61.5 (16.9)	0.02*
Sex (F/M)	194/202 (48.9%/51.1%)	163/232 (41.3%/58.7%)	357/434 (45.1%/54.9%)	0.03**
Average metastases number (SD)	20.9 (27.4)	18.1 (29.7)	19.5 (28.6)	0.162*
Average number of clusters (SD)	5.4 (4.5)	5.0 (4.6)	5.2 (4.6)	0.29*
Average number of metastases in a cluster (SD)	3.9 (4.4)	3.0 (4.7)	3.8 (4.6)	0.01*
Average Linear/parallel Ratio (SD)	0.46 (0.66)	0.22 (0.78)	0.34 (0.74)	4.3 × 10^–6^*

*Two-sided *t*-test

**Fisher exact test

**Fig. 2:**
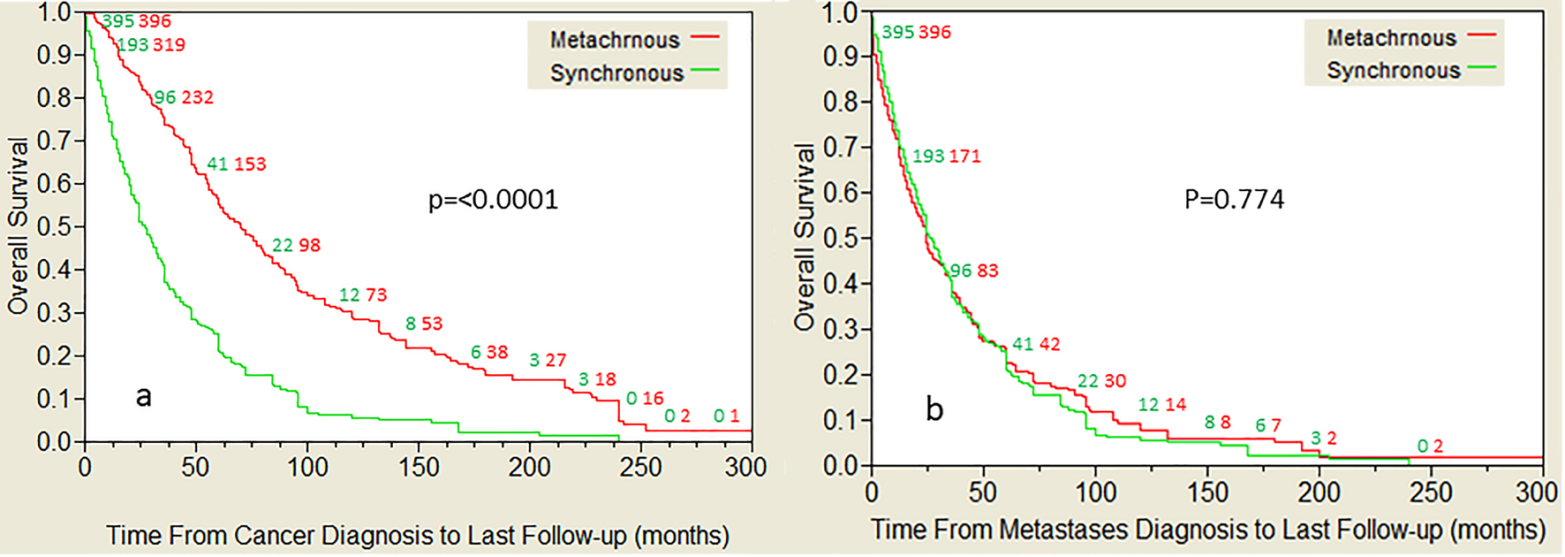
Overall survival of patients with synchronous or metachronous metastases calculated from the time of diagnosis of the primary tumor (**a**), or from the time of diagnosis of metastatic disease (**b**)

During follow-up, 608 patients (76.9%) died. In 601 patients (98.8%), death was disease specific. Kaplan–Meier curves for OS are presented in Fig. 2. When plotted from the time of cancer diagnosis (Fig. 2a), median OS of patients with MM and SM were 69 months and 26 months respectively (*p* < 0.0001). When plotted from the time of metastases diagnosis (Fig. 2b), the curves are virtually identical with a median OS of 23 months in patients with MM and 26 months with SM (*p* = 0.774). OS curves divided by tumor type are available in supplementary material S4.

The number of metastases and the number metastasis clusters for patients with MM and SM were similar. However, the average number of metastases in a cluster in the MM group was 3 (SD 4.7) compared to 3.9 (SD 4.4) in patients with SM (*p* = 0.01).

**Fig. 3:**
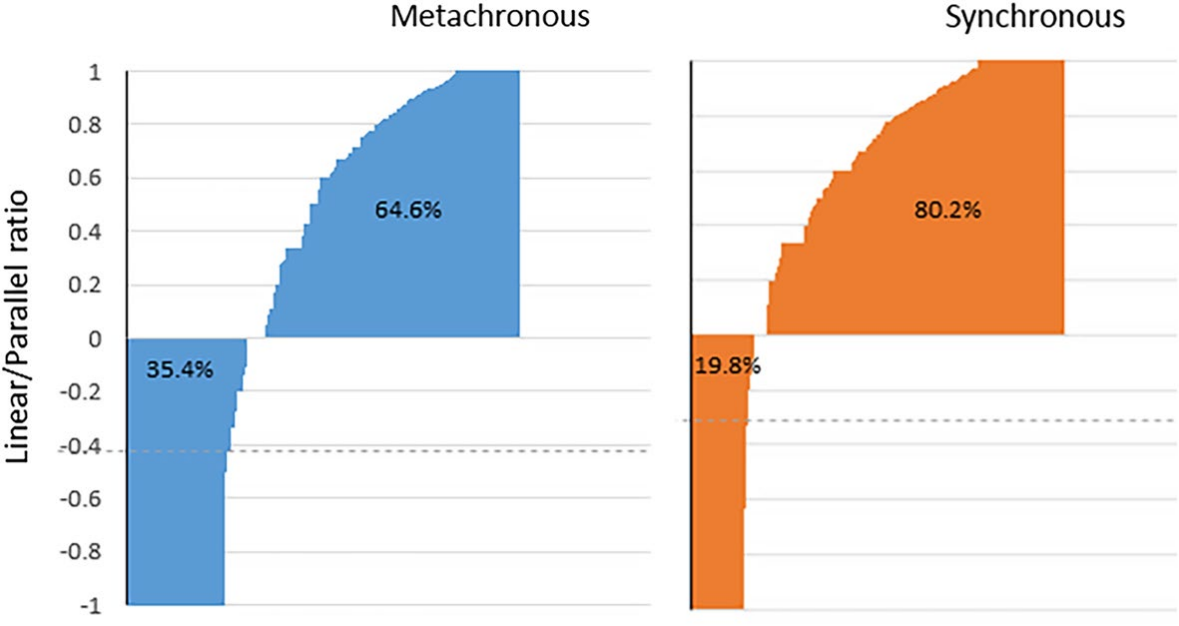
The linear/parallel ratio (LPR) of all patients with metastatic disease. LPR ≤ 0 suggests dominance of the parallel pathway and LPR > 0 linear dominance

The LPR levels of all patients are presented in Fig. 3. A parallel spread LPR ≤ 0 was found in 35.4% of the patients with MM compared to 19.8% of the patients with SM (*p* < 0.00001). The difference in LPR in absolute numbers between MM and SM was also highly significant (*p* < 0.00001).

## Discussion

Metastases can present synchronously, along with the primary tumor or metachronously. MM are detected by a surveillance protocol after treatment of the localized disease. Thus, it is expected that they would be found at an early stage, be smaller and fewer compared to SM, and that patients with MM would have better prognosis.

In the current study, of almost 800 patients with 15,427 lung metastases, we compared the demographics, clonal origin, and prognosis of patients with SM and MM, in an attempt to determine whether SM and MM differ only in lead time or stem from different biological processes. It was found that the median lead-time from diagnosis of the primary tumor to MM was 27 months (IQR 12, 27). When OS was calculated from the time of cancer diagnosis (Fig. 2a), median OS of patients with MM and SM were 69 months and 26 months respectively (*p* < 0.0001), but when OS is calculated from the time of metastases diagnosis (Fig. 2b), the curves were virtually identical with median OS of 23 months in patients with MM and 26 months with SM (*p* = 0.774).

The OS of patients with MM and SM is, therefore remarkably similar. Most studies comparing SM and MM showed similar findings or minor benefit to patients with MM (Slesser et al. 2013; Mitry et al. 2010; Bokhorn et al. 2008; Nozawa et al. 2012; Jeong et al. 2017; Tsai et al. 2007; Mekenkamp et al. 2010; Reboux et al. 2022; Kumar et al. 2014; Colloca et al. 2020; Ghiringhelli et al. 2014; Dawood et al. 2010; Khanfir et al. 2013). How is possible to explain these disappointing findings? We suggest that differences in demography and clonal origin balance the benefit of early diagnosis.

In the current study, patients with MM were found to be older by more than 2 years (*p* = 0.02) and with higher percentage of males (58.7% vs 51.1%, *p* = 0.03) compared to patients with SM. Both parameters are known risk factors for developing lung metastases in patients with CR cancer (Liang et al. 2021). LPR, which provides a glimpse into the dissemination pattern adds more information. LPR ≤ 0, suggesting dominance of the parallel route, was found in 35.4% of patients with MM compared to 19.8% of the patients with SM (*p* < 0.00001). This was also true in absolute (average LPR of 0.46 in SM Vs. 0.22 in MM, *p* = 4.3 × 10^–6^).

This implies that the linear dissemination route is dominant in both types of metastases, but the parallel route is more commonly employed by MM. This is further supported by the smaller number of metastases in each cluster (average of 3.9 metastases in SM and 3 in MM, *p* = 0.01) and is in concordance with most of the genomic work in the field. Due to tissue availability, almost all studies in this field focused on comparisons of primary CR cancer to its liver metastases. Many (but not all) studies support different clonal origins of MM and SM. The molecular studies in metastases progression were done using a variety of methodologists and were rarely validated by further studies. Additionally, as stated before, the situation is complicated by the different definitions of MM and SM used by different authors (Slesser et al. 2013). A brief summary of the important genomic findings follows:

1. In liver SM of CR cancer, the expression of VEGF, CXCR4, COX2 mRNA, TGF-α, and ISR1 was found to be significantly higher in SM compared to MM, while that of Cyclin E, CCR6 and FAK significantly lower. The expression of CD83 and EGFR mRNA was found higher in MM. No differences were found in the expressions of Ki-67, TP, CD31, CD34, c-erb-2 and ZEB2 (Slesser et al. 2013).
2. ATM, KIT, PIK3CA, SMAD4 were more commonly mutated in SM and FBXW7, SMO, STK11 in MM suggesting that SM and MM follow different trajectories of metastatic progression (Kovaleva et al. 2016).
3. In SM there is a higher incidence of venous invasion and overexpression COX-2, while in MM there is greater angiogenesis and higher number of mature dendritic cells (CD83 +) at the invasion margins (Tan and Ooi 2010).
4. Comparison of primary tumors and MM using deep sequencing of DNA showed substantial differences pointing toward parallel progression (Vermaat et al. 2012).
5. Whole exome sequencing and copy number analysis showed that about half of the metastases had clonal origin similar to the primary tumors (linear spread) and half were genetically distinct (parallel spread) (Lee et al. 2014).
6. In another whole exome sequencing study of primary and metastatic tumors, it was found that all metastases inherited multiple genetically distinct sub-clones from the primary tumor, supporting the notion of polyclonal (parallel) seeding (Wei et al. 2017).
7. In contrast to the other studies, comparison of mutation profile of matched primary and metastatic tumors showed more private mutations in SM compared to MM (Brannon et al. 2014).
8. Also, in contrast to the previous studies, analysis of mutational profile of 10 genes in pairs of primary and liver SM and MM patients showed similar mutational profiles indicating linear progress of both types of metastases (Kim et al. 2017).
9. Comparative genomic hybridization (CGH) of 18 pairs of primary and MM showed similar CGH profiles with increased number of chromosomal changes in some of the metastases supporting a linear spread of MM (Jiang et al. 2005).
10. Mutational analysis of liver SM and MM showed more mutations in TP53, NRAS and HRAS in MM and more mutations in APC in SM (Lan et al. 2021)
11. High concordance of KRAS/BRAF mutations status between primary and metastases was found with no difference between SM and MM. However, KRAS/BRAF mutations occur early in CR carcinogenesis and do not provide reliable information regarding later events (Fujiyoshi et al. 2017).
12. Genotype status of primary and metastatic CR cancer showed that MM demonstrates higher frequency of private mutations compared to patients with SM (*p* = 0.0291). In that study, patients with a high percentage of shared primary/metastasis mutations had better prognosis compared to patients with lower percentage (Chatzopoulos et al. 2020).
13. Comparative genomic hybridization of brain SM and MM in renal cell carcinoma showed that losses at 9p and 9q were significantly more common in MM compared SM (*p* = 0.048), suggesting that MM and SM have different pathogenesis (Gutenberg et al. 2014).
14. In stomach cancer, MET status in liver SM correlated with primary tumor status in synchronous metastases but not in MM, supporting linear spread of SM and parallel in MM (Kim et al. 2018).

Another interesting point that can potentially modify tumor growth rate is the potential modifying effect that the primary tumor has on receptivity of host environment, especially in the context of angiogenesis. While the primary tumor is present in all cases of SM it is absent in most MM patients. It was shown that the liver parenchyma adjacent to the SM provides supportive angiogenic environment when the primary tumor is present (high expression of VEGF-A, VEGFR-1, VEGFR-2, and PLGF) making it more permissive for metastasis growth. Additionally, a high Ang-2/Ang-1 ratio was found in adjacent liver parenchyma of SM patients. This supports vascular destabilization, endothelial sprouting, and angiogenesis. This effect is shared by all SM and should not modify the results obtained using the methodology of metastases classification employed in the current study (van der Wal et al. 2012).

Taken together, these results suggest that SM and MM are different conditions, and the parallel route is more common in MM. Despite all this effort, a unifying theory explaining the patho-physiology of SM and MM is absent. Additionally, the cited studies may not reliably reflect the true trajectory of the metastatic disease since analyzed tissues were often taken at different time points which may conceal the modifying/selecting effect of therapy. Moreover, these studies are limited to patients with oligometastatic disease that may not fully represent more advanced conditions.

Parallel spread, that as shown here is more common in MM, generates higher diversity and increased resistance to therapy. Additionally, prior exposure to chemotherapeutic agents in this group may further add to chemoresistance. This is supported by the higher response rate to first line chemotherapy observed in patients with SM compared to MM (Mekenkamp et al. 2010).

This study has several limitations worth mentioning. The conclusions are limited to lung metastases. Application of the model to other metastatic sites should be proved in further studies. The model assumes that similar metastasis diameter equals identical clonal origin. Yet, different proximity to nutrients or to anatomical structures of cells originating in a single clone can modify their growth rate and mimic parallel growth. A later, nevertheless faster growing clone can reach the same size as an earlier but slower one and mimic linear growth.

## Conclusions

Despite different pathogenesis, patients with MM and SM have an almost identical OS. This is most likely due to counterbalancing forces. On one hand, better outcome due to earlier detection in MM. On the other hand, worse outcome due to inferior demography and higher frequency of parallel dissemination. Early diagnosis in patients at risk of developing cancer may decrease the incidence of SM. Better identification of patients at risk of developing MM and aggressive adjuvant or neoadjuvant treatments may eradicate DTCs before they evolve into mature MM.

## Supplementary Information

Below is the link to the electronic supplementary material.

Supplementary file1 (XLS 271 KB)

Supplementary file2 (DOCX 13 KB)

Supplementary file3 (DOCX 25 KB)

Supplementary file4 (TIF 150 KB)

## Data Availability

All data are available in supplementary material S1.

## References

[CR1] Bockhorn M, Frilling A, Frühauf NR, Neuhaus J, Molmenti E, Trarbach T, Malagó M, Lang H, Broelsch CE (2008). Survival of patients with synchronous and metachronous colorectal liver metastases–is there a difference?. J Gastrointest Surg.

[CR2] Brannon AR, Vakiani E, Sylvester BE, Scott SN, McDermott G, Shah RH, Kania K (2014). Comparative sequencing analysis reveals high genomic concordance between matched primary and metastatic colorectal cancer lesions. Genome Biol.

[CR3] Chatzopoulos K, Kotoula V, Koliou GA, Giannoulatou E, Papadopoulou K, Karavasilis V (2020). Genotype-phenotype associations in colorectal adenocarcinomas and their matched metastases. Hum Pathol.

[CR4] Colloca GA, Venturino A, Guarneri D (2020). Different variables predict the outcome of patients with synchronous versus metachronous metastases of colorectal cancer. Clin Transl Oncol.

[CR5] Dawood S, Broglio K, Ensor J, Hortobagyi GN, Giordano SH (2010). Survival differences among women with de novo stage IV and relapsed breast cancer. Ann Oncol.

[CR6] Fouad YA, Aanei C (2017). Revisiting the hallmarks of cancer. Am J Cancer Res.

[CR7] Fujiyoshi K, Yamamoto G, Takahashi A, Arai Y, Yamada M, Kakuta M (2017). High concordance rate of KRAS/BRAF mutations and MSI-H between primary colorectal cancer and corresponding metastases. Oncol Rep.

[CR8] Ghiringhelli F, Hennequin A, Drouillard A, Lepage C, Faivre J, Bouvier AM (2014). Epidemiology and prognosis of synchronous and metachronous colon cancer metastases: a French population-based study. Dig Liver Dis.

[CR9] Gofrit ON, Gofrit B, Roditi Y, Popovtzer A, Frank S, Sosna J, Goldberg SN (2022). Patterns of metastases progression—the linear parallel ratio. PLoS ONE.

[CR10] Gutenberg A, Nischwitz MD, Gunawan B, Enders C, Jung K, Bergmann M, Feiden W (2014). Predictive chromosomal clusters of synchronous and metachronous brain metastases in clear cell renal cell carcinoma. Cancer Genet.

[CR11] Jeong S, Heo JS, Park JY, Choi DW, Choi SH (2017). Surgical resection of synchronous and metachronous lung and liver metastases of colorectal cancers. Ann Surg Treat Res.

[CR12] Jiang JK, Chen YJ, Lin CH, Yu IT, Lin JK (2005). Genetic changes and clonality relationship between primary colorectal cancers and their pulmonary metastases—an analysis by comparative genomic hybridization. Genes Chromosomes Cancer.

[CR13] Khanfir A, Lahiani F, Bouzguenda R, Ayedi I, Daoud J, Frikha M (2013). Prognostic factors and survival in metastatic breast cancer: a single institution experience. Rep Pract Oncol Radiother.

[CR14] Kim KP, Kim JE, Hong YS, Ahn SM, Chun SM, Hong SM (2017). Paired primary and metastatic tumor analysis of somatic mutations in synchronous and metachronous colorectal cancer. Cancer Res Treat.

[CR15] Kim HS, Chon HJ, Kim H, Shin SJ, Wacheck V, Gruver AM (2018). MET in gastric cancer with liver metastasis: the relationship between MET amplification and Met overexpression in primary stomach tumors and liver metastasis. J Surg Oncol.

[CR16] Kovaleva V, Geissler AL, Lutz L, Fritsch R, Makowiec F, Wiesemann S (2016). Spatio-temporal mutation profiles of case-matched colorectal carcinomas and their metastases reveal unique de novo mutations in metachronous lung metastases by targeted next generation sequencing. Mol Cancer.

[CR17] Kumar R, Price TJ, Beeke C, Jain K, Patel G, Padbury R (2014). Colorectal cancer survival: an analysis of patients with metastatic disease synchronous and metachronous with the primary tumor. Clin Colorectal Cancer.

[CR18] Lan YT, Chang SC, Lin PC, Lin CC, Lin HH, Huang SC (2021). Clinicopathological and molecular features between synchronous and metachronous metastases in colorectal cancer. Am J Cancer Res.

[CR19] Lee SY, Haq F, Kim D, Jun C, Jo HJ, Ahn SM, Lee WS (2014). Comparative genomic analysis of primary and synchronous metastatic colorectal cancers. PLoS ONE.

[CR20] Liang X, Cheng Y, Zhou W, Ni J, Li Y, Feng G (2021). Systematic pan-cancer population-based analysis reveals the incidence and prognosis of lung metastases at diagnosis. J Oncol.

[CR21] Mekenkamp LJ, Koopman M, Teerenstra S, van Krieken JH, Mol L, Nagtegaal ID, Punt CJ (2010). Clinicopathological features and outcome in advanced colorectal cancer patients with synchronous vs metachronous metastases. Br J Cancer.

[CR22] Mitry E, Guiu B, Cosconea S, Jooste V, Faivre J, Bouvier AM (2010). Epidemiology, management and prognosis of colorectal cancer with lung metastases: a 30-year population-based study. Gut.

[CR23] Nozawa H, Sunami E, Nakajima J, Nagawa H, Kitayama J (2011). Synchronous and metachronous lung metastases in patients with colorectal cancer: a 20-year monocentric experience. Exp Ther Med.

[CR24] Reboux N, Jooste V, Goungounga J, Robaszkiewicz M, Nousbaum JB, Bouvier AM (2022). Incidence and survival in synchronous and metachronous liver metastases from colorectal cancer. JAMA Netw Open.

[CR25] Slesser AA, Georgiou P, Brown G, Mudan S, Goldin R, Tekkis P (2013). The tumour biology of synchronous and metachronous colorectal liver metastases: a systematic review. Clin Exp Metastasis.

[CR26] Stoecklein NH, Klein CA (2010). Genetic disparity between primary tumours, disseminated tumour cells, and manifest metastasis. Int J Cancer.

[CR27] Tan EK, Ooi LL (2010). Colorectal cancer liver metastases—understanding the differences in the management of synchronous and metachronous disease. Ann Acad Med Singap.

[CR28] Tsai MS, Su YH, Ho MC, Liang JT, Chen TP, Lai HS, Lee PH (2007). Clinicopathological features and prognosis in resectable synchronous and metachronous colorectal liver metastasis. Ann Surg Oncol.

[CR29] van der Wal GE, Gouw AS, Kamps JA, Moorlag HE, Bulthuis ML, Molema G, de Jong KP (2012). Angiogenesis in synchronous and metachronous colorectal liver metastases: the liver as a permissive soil. Ann Surg.

[CR30] Vermaat JS, Nijman IJ, Koudijs MJ, Gerritse FL, Scherer SJ, Mokry M (2012). Primary colorectal cancers and their subsequent hepatic metastases are genetically different: implications for selection of patients for targeted treatment. Clin Cancer Res.

[CR31] Wei Q, Ye Z, Zhong X, Li L, Wang C, Myers RE (2017). Multiregion whole-exome sequencing of matched primary and metastatic tumors revealed genomic heterogeneity and suggested polyclonal seeding in colorectal cancer metastasis. Ann Oncol.

