# Supplementary material for: The different clonal origins of metachronous and synchronous metastases
Source: J Cancer Res Clin Oncol. 2023 Jun 20;149(13):11085–92. doi: 10.1007/s00432-023-05007-3 (PMC10465669; doi:10.1007/s00432-023-05007-3)
Supplement: Supplementary file 3 — Supplementary file3 (DOCX 25 KB) [file 432_2023_5007_MOESM3_ESM.docx]

Supplementary Material 3. Demographic and metastases parameters broken by tumor type.

Bladder

| p | Total | Metachronous | Synchronous | Type |
| --- | --- | --- | --- | --- |
| 0.29* | 71.9 (8.9) | 70.8 (7.9) | 73.9 (10.7) | Average age at diagnosis of cancer(SD) |
| 0.82* | 73.5 (8.6) | 73.3 (7.5) | 73.9 (10.7) | Average age at diagnosis of metastases (SD) |
| 0.22** | 7/45(13.5%/86.5%) | 3/31 (8.9%/91.1%) | 4/14 (22.2%/ 77.8%) | Sex (F/M) |
| 0.05* | 18.2 (29.8) | 22.6 (35.8) | 9.8 (7.7) | Average metastases number (SD) |
| 0.15* | 5.5 (5.2) | 6.2 (6.1) | 4.3 (2.7) | Average number of clusters (SD) |
| 0.23* | 2.6 (2.3) | 2.8 (2.7) | 2.2 (1.0) | Average number of metastases in a cluster (SD) |
| 0.49* | 0.22(0.78) | 0.16 (0.79) | 0.32 (0.79) | Average Linear/parallel Ratio (SD) |

*Two sided t-test **Fisher exact test

Breast

| p | Total | Metachronous | Synchronous | Type |
| --- | --- | --- | --- | --- |
| 0.96* | 56.1 (15.7) | 56.2 (15.8) | 56.1 (15.7) | Average age at diagnosis of cancer(SD) |
| 0.04* | 59.3 (16.8) | 62.4 (16.4) | 56.1 (15.7) | Average age at diagnosis of metastases (SD) |
| 0.27** | 101/7(93.5%/6.5%) | 53/2 (96.4%/3.6%) | 48/5 (90.6%/9.4%) | Sex (F/M) |
| 0.34* | 14.3 (19.1) | 16.2 (19.4) | 16.0 (18.7) | Average metastases number (SD) |
| 0.46* | 5.0 (3.5) | 4.8 (3.3) | 5.3 (3.7) | Average number of clusters (SD) |
| 0.25* | 2.7 (1.9) | 2.5 (2.2) | 2.9 (1.7) | Average number of metastases in a cluster (SD) |
| 0.002* | 0.32 (0.68) | 0.13 (0.75) | 0.56 (0.55) | Average Linear/parallel Ratio (SD) |

Colorectum

| p | Total | Metachronous | Synchronous | Type |
| --- | --- | --- | --- | --- |
| 0.04* | 60.5 (14.4) | 62.5 (13.1) | 58.8 (15.3) | Average age at diagnosis of cancer(SD) |
| 0.0006* | 61.8 (14.7) | 65.2 (13.4) | 58.8 (15.3) | Average age at diagnosis of metastases (SD) |
| 0.69** | 105/135(43.7%/56.3%) | 47/65(41.9%/58.1%) | 58/70 (45.3%/ 54.7%) | Sex (F/M) |
| 0.48* | 21.1 (31.5) | 19.6 (31.9) | 22.4 (31.1) | Average metastases number (SD) |
| 0.036* | 5.5 (4.7) | 4.1 (4.7) | 6.1 (4.7) | Average number of clusters (SD) |
| 0.67* | 3.2 (5.3) | 3.4 (7.3) | 3.1 (2.6) | Average number of metastases in a cluster (SD) |
| 0.053* | 0.28 (0.74) | 0.18 (0.79) | 0.37 (0.69) | Average Linear/parallel Ratio (SD) |

Kidney

| p | Total | Metachronous | Synchronous | Type |
| --- | --- | --- | --- | --- |
| 0.56* | 62.4 (12.5) | 63.1 (11.0) | 61.2 (11.8) | Average age at diagnosis of cancer(SD) |
| 0.056* | 65.3 (13.3) | 67.9 (11.8) | 61.2 (11.8) | Average age at diagnosis of metastases (SD) |
| 0.25** | 17/69(24.6%/75.4%) | 8/34(19.0%/81.0%) | 9/18 (39.1%/ 60.9%) | Sex (F/M) |
| 0.02* | 18.5 (28.9) | 11.7 (23.9) | 29.2 (23.9) | Average metastases number (SD) |
| 0.036* | 4.1 (3.9) | 3.3 (3.2) | 5.4 (3.2) | Average number of clusters (SD) |
| 0.025* | 3.8 (4.0) | 2.8 (3.7) | 5.2 (3.3) | Average number of metastases in a cluster (SD) |
| 0.018* | 0.39 (0.78) | 0.23 (0.86) | 0.65 (0.57) | Average Linear/parallel Ratio (SD) |

Melanoma

| p | Total | Metachronous | Synchronous | Type |
| --- | --- | --- | --- | --- |
| 0.09* | 61.2 (15.1) | 59.3 (17.3) | 65.6 (10.6) | Average age at diagnosis of cancer(SD) |
| 0.51* | 63.3 (15.3) | 62.6 (17.3) | 65.6 (10.6) | Average age at diagnosis of metastases (SD) |
| 0.22** | 30/48(38.5%/61.5%) | 16/32(33.3%/66.7%) | 14/14 (50.0%/ 50.0%) | Sex (F/M) |
| 0.61* | 18.9 (29.7) | 20.0 (34.5) | 17.0 (18.1) | Average metastases number (SD) |
| 0.14* | 5.5 (4.5) | 4.8 (3.9) | 6.5 (5.3) | Average number of clusters (SD) |
| 0.27* | 2.5 (2.4) | 2.7 (2.8) | 2.2 (1.3) | Average number of metastases in a cluster (SD) |
| 0.36* | 0.16(0.78) | 0.09 (0.81) | 0.26 (0.73) | Average Linear/parallel Ratio (SD) |

Prostate

| p | Total | Metachronous | Synchronous | Type |
| --- | --- | --- | --- | --- |
| 0.69* | 69.9 (10.3) | 70.8 (6.9) | 69.4 (11.8) | Average age at diagnosis of cancer(SD) |
| 0.11* | 71.5 (10.8) | 75.2 (7.8) | 69.4 (11.8) | Average age at diagnosis of metastases (SD) |
| 1** | 30/48(38.5%/61.5%) | 0/12(33.3%/66.7%) | 0/22 (0.0%/ 100.0%) | Sex (F/M) |
| 0.77* | 18.1 (27.4) | 20.6 (42.0) | 16.8 (15.8) | Average metastases number (SD) |
| 0.33* | 2.7 (2.2) | 2.2 (1.9) | 3 (2.3) | Average number of clusters (SD) |
| 0.23* | 6.5 (7.5) | 5.7 (5.8) | 6.9 (8.4) | Average number of metastases in a cluster (SD) |
| 0.75* | 0.76 (0.49) | 0.79 (0.58) | 0.74 (0.45) | Average Linear/parallel Ratio (SD) |

Sarcoma

| p | Total | Metachronous | Synchronous | Type |
| --- | --- | --- | --- | --- |
| 0.94* | 51.7 (19.0) | 51.9 (18.7) | 51.6 (19.7) | Average age at diagnosis of cancer(SD) |
| 0.5* | 53.2 (18.8) | 54.5 (18.1) | 51.6 (19.7) | Average age at diagnosis of metastases (SD) |
| 1** | 42/38(52.5%/47.5%) | 19/27(41.3%/58.7%) | 23/11 (67.6%/ 32.4%) | Sex (F/M) |
| 0.99* | 19.5 (18.8) | 19.9 (20.3) | 19.5 (16.7) | Average metastases number (SD) |
| 0.82* | 8.3 (5.7) | 8.2 (5.0) | 8.5 (6.5) | Average number of clusters (SD) |
| 0.34* | 2.1 (1.1) | 2 (1.2) | 2.3 (1.0) | Average number of metastases in a cluster (SD) |
| 0.29* | 0.19 (0.69) | 0.13 (0.71) | 0.29 (0.71) | Average Linear/parallel Ratio (SD) |

Thyroid

| p | Total | Metachronous | Synchronous | Type |
| --- | --- | --- | --- | --- |
| 0.0006* | 31.1 (15.4) | 53.0 (15.6) | 68.2 (11.5) | Average age at diagnosis of cancer(SD) |
| 0.02* | 63.8 (14.0) | 58.8 (15.2) | 68.2 (11.5) | Average age at diagnosis of metastases (SD) |
| 0.56** | 42/38(52.5%/47.5%) | 9/13(40.9%/59.1%) | 13/12 (52.0%/ 48.0%) | Sex (F/M) |
| 0.07* | 34.3 (44.3) | 27.7 (87.8) | 49.9 (49.9) | Average metastases number (SD) |
| 0.7* | 4.6 (3.1) | 4.4 (3.4) | 4.7 (2.9) | Average number of clusters (SD) |
| 0.05* | 7.1 (7.6) | 4.9 (5.6) | 9.11 (8.7) | Average number of metastases in a cluster (SD) |
| 0.4* | 0.75 (0.40) | 0.79 (0.29) | 0.71 (0.47) | Average Linear/parallel Ratio (SD) |

Ewing

| p | Total | Metachronous | Synchronous | Type |
| --- | --- | --- | --- | --- |
| 0.96* | 17.9 (8.9) | 17.9 (4.7) | 18.1 (12.6) | Average age at diagnosis of cancer(SD) |
| 0.65* | 19.2 (9.2) | 20.1 (5.4) | 18.1 (12.6) | Average age at diagnosis of metastases (SD) |
| 1** | 42/38(52.5%/47.5%) | 6/6(50.0%/50.0%) | 5/5 (50.0%/ 50.0%) | Sex (F/M) |
| 0.35* | 19.3 (33.5) | 25.2 (43.6) | 12.3 (14.2) | Average metastases number (SD) |
| 0.08* | 5.8 (6.0) | 7.8 (7.4) | 3.5 (2.7) | Average number of clusters (SD) |
| 0.29* | 2.9 (3.4) | 2.2 (1.6) | 3.9 (4.7) | Average number of metastases in a cluster (SD) |
| 0.91* | 0.05 (0.91) | 0.03 (0.92) | 0.05 (0.91) | Average Linear/parallel Ratio (SD) |

Pancreas

| p | Total | Metachronous | Synchronous | Type |
| --- | --- | --- | --- | --- |
| 0.62* | 66.7 (12.3) | 63.0 (11.5) | 67.0 (12.4) | Average age at diagnosis of cancer(SD) |
| 0.82* | 66.8 (12.3) | 65.2 (12.2) | 67.0 (12.4) | Average age at diagnosis of metastases (SD) |
| 0.59** | 20/23(46.5%/53.5%) | 2/1(66.7%/33.3%) | 18/22 (45.0%/ 55.0%) | Sex (F/M) |
| 0.004* | 18.4 (19.9) | 5.0 (4.3) | 19.4 (20.3) | Average metastases number (SD) |
| 0.002* | 3.5 (3.3) | 1.3 (0.58) | 3.6 (3.4) | Average number of clusters (SD) |
| 0.77* | 5.6 (4.9) | 4.7 (4.7) | 5.6 (5.0) | Average number of metastases in a cluster (SD) |
| 0.61* | 0.71 (0.59) | 0.33 (0.92) | 0.74 (0.55) | Average Linear/parallel Ratio (SD) |

Stomach

| p | Total | Metachronous | Synchronous | Type |
| --- | --- | --- | --- | --- |
| 0.15* | 64.4 (15.9) | 72.3 (15.1) | 60.4 (15.1) | Average age at diagnosis of cancer(SD) |
| 0.10* | 64.8 (16.0) | 73.6 (14.7) | 60.4 (15.1) | Average age at diagnosis of metastases (SD) |
| 0.53** | 2/16(11.1%/89.9%) | 0/6(0%/100%) | 2/10 (16.7%/ 83.3%) | Sex (F/M) |
| 0.62* | 6.6 (1.1) | 8.0 (7.9) | 5.92 (7.97) | Average metastases number (SD) |
| 0.86* | 3.2 (2.4) | 1.7 (0.82) | 1.75 (0.82) | Average number of clusters (SD) |
| 0.28* | 3.2 (2.4) | 4.0 (2.1) | 2.7 (2.1) | Average number of metastases in a cluster (SD) |
| 0.06* | 0.64 (0.76) | 0.45 (0.89) | 1.0 (0) | Average Linear/parallel Ratio (SD) |
