# Supplementary figures and images for: The different clonal origins of metachronous and synchronous metastases
Source: J Cancer Res Clin Oncol. 2023 Jun 20;149(13):11085–92. doi: 10.1007/s00432-023-05007-3 (PMC10465669; doi:10.1007/s00432-023-05007-3)

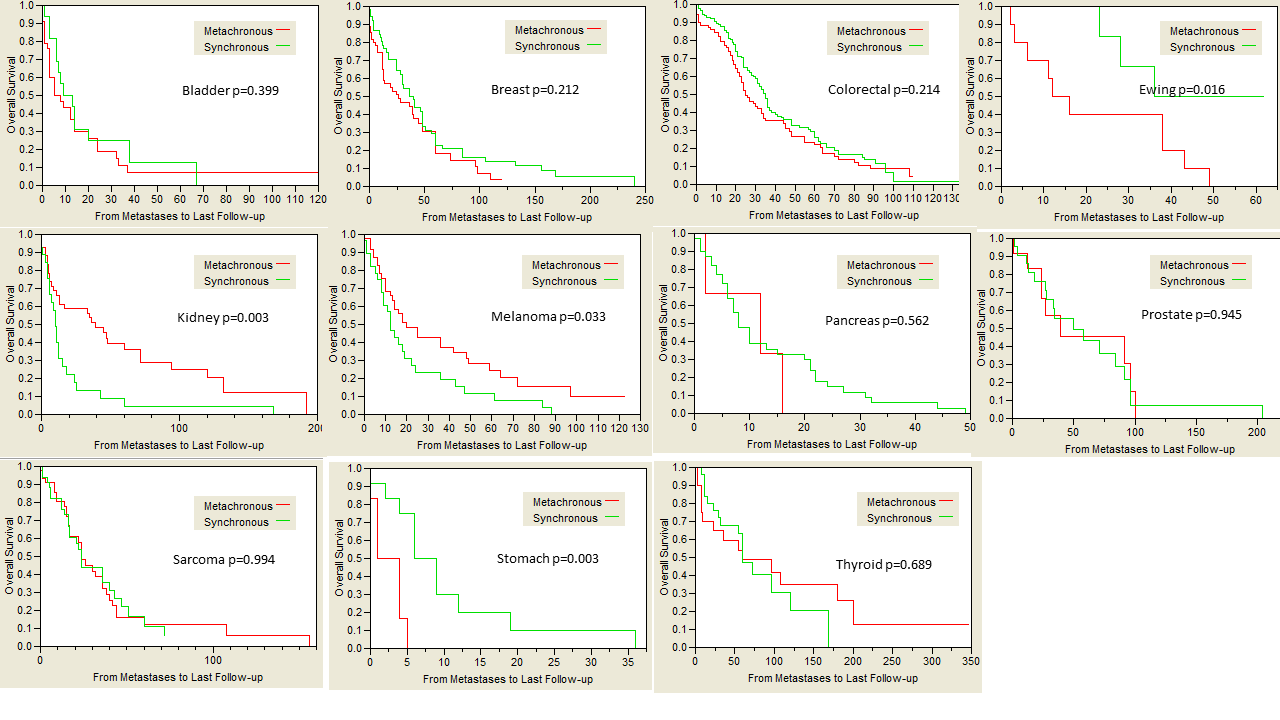

Supplement: Supplementary file 4 — Supplementary file4 (TIF 150 KB) [file 432_2023_5007_MOESM4_ESM.tif]
